# Human embryonic stem cell-derived mesenchymal stromal cells ameliorate collagen-induced arthritis by inducing host-derived indoleamine 2,3 dioxygenase

**DOI:** 10.1186/s13075-016-0979-0

**Published:** 2016-04-01

**Authors:** Elena Gonzalo-Gil, María J. Pérez-Lorenzo, María Galindo, Rafael Díaz de la Guardia, Belén López-Millán, Clara Bueno, Pablo Menéndez, José L. Pablos, Gabriel Criado

**Affiliations:** Inflammatory and Autoimmune Diseases Group, Hospital 12 de Octubre Research Institute, Madrid, Spain; Rheumatology Department, Hospital 12 de Octubre, Madrid, Spain; Josep Carreras Leukemia Research Institute, School of Medicine, University of Barcelona, Barcelona, Spain; Institució Catalana de Recerca i Estudis Avançats (ICREA), Barcelona, Spain; Inflammatory and Autoimmune Diseases Group, Hospital 12 de Octubre Research Center, Avenida de Córdoba s/n. 28041, Madrid, Spain

**Keywords:** Arthritis, Human embryonic stem cell-derived mesenchymal stromal cells, T cells, Cytokines, Indoleamine 2,3 dioxygenase

## Abstract

**Background:**

The immunosuppressive and anti-inflammatory properties of mesenchymal stromal cells (MSC) have prompted their therapeutic application in several autoimmune diseases, including rheumatoid arthritis. Adult MSC are finite and their clinical use is restricted by the need for long-term expansion protocols that can lead to genomic instability. Inhibition of Smad2/3 signaling in human pluripotent stem cells (hPSC) provides an infinite source of MSC that match the phenotype and functional properties of adult MSC. Here, we test the therapeutic potential of hPSC-MSC of embryonic origin (embryonic stem cell-derived mesenchymal stromal cells, hESC-MSC) in the experimental model of collagen-induced arthritis (CIA).

**Methods:**

CIA was induced in DBA/1 mice by immunization with type II collagen (CII) in Complete Freund’s Adjuvant (CFA). Mice were treated with either a single dose (10^6^ cells/mouse) of hESC-MSC on the day of immunization (prophylaxis) or with three doses of hESC-MSC every other day starting on the day of arthritis onset (therapy). Arthritis severity was evaluated daily for six weeks and ten days, respectively. Frequency of Treg (FoxP3^+^), Th1 (IFNγ^+^) and Th17 (IL17^+^) CD4^+^ T cells in inguinal lymph nodes (ILN) was quantified by flow cytometry. Serum levels of anti-CII antibodies were determined by ELISA. Detection of hESC-MSC and quantification of murine and human indoleamine 2,3 dioxygenase (IDO1) expression was performed by quantitative real-time PCR. Statistical differences were analyzed by ANOVA and the Mann-Whitney *U* test.

**Results:**

Administration of hESC-MSC to mice with established arthritis reduced disease severity compared to control-treated mice. Analysis of CD4 T cell populations in treated mice showed an increase in FoxP3^+^ Treg and IFNγ^+^ Th1 cells but not in Th17 cells in the ILN. Anti-CII antibody levels were not affected by treatment. Migration of hESC-MSC to the ILN in treated mice was associated with the induction of murine IDO1.

**Conclusion:**

Treatment with hESC-MSC ameliorates CIA by inducing IFNγ^+^ Th1 cells and IDO1 in the host. Thus, hESC-MSC can provide an infinite cellular source for treatment of rheumatoid arthritis.

## Background

Mesenchymal stromal cells (MSC) are multipotent progenitor cells that can differentiate into lineages of mesenchymal tissues, including bone, cartilage, and adipose tissues [1]. MSC have been isolated from a variety of adult tissues, such as bone marrow, skeletal muscle, synovium, dental pulp, liver, muscle, brain, placenta, bone, umbilical cord, and adipose tissue [2, 3]. MSC lack immunogenicity, display robust immunosuppressive and anti-inflammatory properties, promote angiogenesis, and reduce apoptosis, a set of features that have prompted their wide use in regenerative medicine [4–6]. MSC play a regulatory role on immune responses by producing soluble factors, including transforming growth factor (TGF)-β, hepatocyte growth factor, nitric oxide, hemoxygenase, IL6, prostaglandin E2, and HLA-G [7–9]. In addition, MSC can produce high levels of indoleamine 2,3 dioxygenase (IDO1), a tryptophan catabolizing enzyme that mediates immune tolerance by limiting the availability of the essential amino acid tryptophan and generating toxic metabolites for T cells [10].

Due to their immunosuppressive properties and low immunogenicity, MSC have been proposed as therapeutic agents for autoimmune diseases such as multiple sclerosis, autoimmune diabetes, systemic lupus erythematosus, and rheumatoid arthritis (RA) [11–13]. Recently, several studies have investigated the therapeutic role of MSC in the treatment of collagen induced-arthritis (CIA), a well-characterized model of chronic arthritis [14, 15]. One recent study has demonstrated that adult bone marrow allogeneic MSC migrate to the affected paws and reduce the severity of arthritis in CIA [16]. It has also been shown that human umbilical cord-derived and adipose-tissue-derived MSC reduce the incidence and severity of arthritis in mice by inhibiting proinflammatory cytokines and inducing activation of regulatory T cells (Treg) [17–19]. Adult MSC are finite and their potential clinical use requires long-term expansion protocols, which might be associated with in vitro acquired genomic instability and reactivation of immunogenicity [20]. Recently, through inhibition of Smad-2/3 signaling, MSC were reproducibly derived from human pluripotent stem cells (hPSC) spanning both embryonic stem cells (hESC) and induced pluripotent stem cells (iPSC) [6, 21]. Human PSC represent an infinite source for MSC, and hPSC-MSC display an identical phenotype, functional properties, and in vitro and in vivo immunosuppressive and anti-inflammatory features compared to adult bone-marrow-derived or cord-blood-derived MSC, as demonstrated in experimental models of acute inflammation in vivo when administered prophylactically [6]. These findings prompted us to investigate the potential therapeutic use of hESC-MSC in the setting of the chronic inflammatory model of CIA. Administration of hESC-MSC in mice with established disease reduced the severity of arthritis and increased the frequency of Treg and T helper (Th)1 cells without affecting Th17 cells. hESC-MSC administered to mice with CIA migrated to draining lymph nodes and induced IDO1 expression.

## Methods

### Human ESC-derived MSC

hESC-MSC were differentiated in vitro and phenotypically/functionally characterized as previously described [6]. hESC-MSC were cultured in Advanced-DMEM with 10 % FCS, 1 % Glutamax, and 1 % penicillin/streptomycin and were allowed to expand and reach nearly 100 % confluence. hESC-MSC at passage ≤10 were used for the in vivo experiments.

### Mice

DBA/1OlaHsd mice were purchased from Harlan laboratories and maintained under specific pathogen-free conditions in the Animal Facility of the Hospital 12 de Octubre. All animal experiments followed institutional guidelines and were approved by the Hospital 12 de Octubre Animal Welfare Committee.

### CIA induction and treatment

Ten-week-old male mice were immunized by intradermal injection at the base of the tail with 200 μg of chicken CII in Complete Freund’s Adjuvant (CFA), as previously described [22]. Mice were treated with 10^6^ hESC-MSC or PBS (control mice) intraperitoneally (i.p.) on the day of immunization and euthanized 6 weeks later (prophylactic protocol) or treated on days 1, 3 and 5 starting on the day of arthritis onset and killed 10 days later (therapeutic protocol). Joint inflammation was measured daily starting on the day of arthritis onset, using a 0–10-mm microcalliper (Mitutoyo, Japan). The clinical severity of arthritis was graded daily for each paw by two independent observers using the following scoring system: 0 = normal, 1 = slight swelling and erythema, 2 = pronounced edematous swelling, and 3 = stiffness of the joint, yielding a maximum score of 12 per mouse. Disease severity was expressed as the mean clinical score ± the standard error of the mean (SEM) per group of treatment [22]. Seven to ten mice per group were included in each experiment.

### Histological joint inflammation and cartilage damage

Paws were removed and fixed overnight in 4 % formaldehyde, decalcified with Immunocal (Decal Chemical Corp, NY, USA) and embedded in paraffin. Sections (5 μm) were prepared and stained with hematoxylin-eosin for assessment of inflammation and histological damage.

### CII-specific responses

Serum was collected from mice with CIA after 10 days of treatment and levels of anti-collagen IgG1 and IgG2a antibodies were assessed by ELISA as previously described [22]. A standard curve was generated for each assay by including serial dilutions of a reference sample of pooled sera from arthritic mice. Relative antibody units (AU) were obtained for each sample by reference to the standard curve.

To address CII-specific responses in draining inguinal lymph nodes (ILN), single cell suspensions were cultured in the presence of 50 μg/ml CII. The specific proliferative response to CII was determined by the incorporation of WST-1 (Roche, Mannheim, Germany) after 72 hours. Cytokine production in culture supernatants collected after 48 hours was measured by ELISA (interferon (IFN)γ and IL17, ELISAMax Standard Kits, Biolegend, CA, USA.) and flow cytometry (IL2, mouse Th1/Th2/Th17 13plex kit, Affimetrix, CA, USA).

### Flow cytometry

Single cell suspensions from ILN were stimulated with 20 ng/ml PMA (Sigma-Aldrich) and 1 μg/ml ionomycin (Sigma-Aldrich) in the presence of 10 μg/ml brefeldin A (Sigma-Aldrich) for 4 hours at 37 °C, 5 % CO_2_. Cells were stained with anti-CD4-PerCP antibody (clone RM4-5, BD Biosciences, San Diego, CA, USA), permeabilized with 0.5 % saponin and stained with anti-IFNγ-fluorescein isothiocyanate (FITC) (clone XMG1.2, BD Biosciences) and anti-IL17-PE (clone TC11-18H10, MiltenyiBiotec, Bergisch Gladbach, Germany) antibodies.

To detect Treg, single cell suspensions from ILN were stained with anti-CD4-PerCP and anti-CD25-FITC (clone 7D4, BD Biosciences) antibodies, fixed, permeabilized and stained with anti-Foxp3-PE antibody (cloneMF-23, BD Biosciences) following the manufacturer’s instructions. Isotype controls were included in all experiments to adjust the background signal. Flow cytometer analysis was performed in a FACSCalibur instrument and analyzed with Cell Quest Pro software (BD Biosciences).

### Quantitative real-time polymerase chain reaction (RT-qPCR)

ILN and paws were snap frozen in liquid nitrogen, pulverized, and total RNA extracted using TRI reagent (Sigma-Aldrich, Madrid, Spain): 1 μg was used for first-strand complementary DNA (cDNA) synthesis with the High Capacity cDNA Transcription Kit (Applied Biosystems; Warrington, UK) and quantitative RT-qPCR was performed on an Applied Biosystems 7500 Fast Real-Time PCR System using Power SYBR Green PCR Master Mix. Data obtained were normalized to a standard curve obtained by serial dilution of a template cDNA and expressed as the ratio between the target gene and glyceraldehyde-3-phosphate dehydrogenase (*GAPDH*). Primer sequences were as follows: Human IDO1(hIDO1): forward: 5′-GCCCTTCAAGTGTTTCAC-3′, reverse: 5′-CCAGCCAGACAAATATATGCGA-3′; mouse IDO (mIDO1): forward: 5′-CAAAGCAATCCCCACTGTATCC-3′, reverse: 5′-ACAAAGTCACGCATCCTCTTAAA-3′; human GAPDH (hGAPDH): forward: 5′-TGTTGCCATCAATGACCCCTT-3′, reverse: 5′-CTCCACGACGTACTCAGCG-3′; mouse GAPDH: (mGAPDH) forward: 5′-CATGGCCTTCCGTGTTCCTA-3′, reverse: 5′-GCGGCACGTCAGATCCA-3′; HLA-C: forward: 5′-GCAGCACGAGGGGCTGCAAG-3′; reverse: 5′-TTGCTGCACGCAGCCTGAGA-3′.

### Statistical analysis

Descriptive analysis of each experiment was performed to compare the incidence and severity of the disease. The impact of hESC-MSC on the clinical scores for mice with CIA and the course of disease was analyzed by two-way analysis of variance (ANOVA) for repeated measures. Differences between groups were compared using the Mann-Whitney *U* test for two independent samples or one-way ANOVA using the Kruskal-Wallis non-parametric test, as required. Analysis was performed using GraphPad Prism software (version 6.0, Graph Pad; CA, USA). *P* values below 0.05 were considered statistically significant.

## Results

### hESC-MSC ameliorate established collagen-induced arthritis

**Fig. 1:**
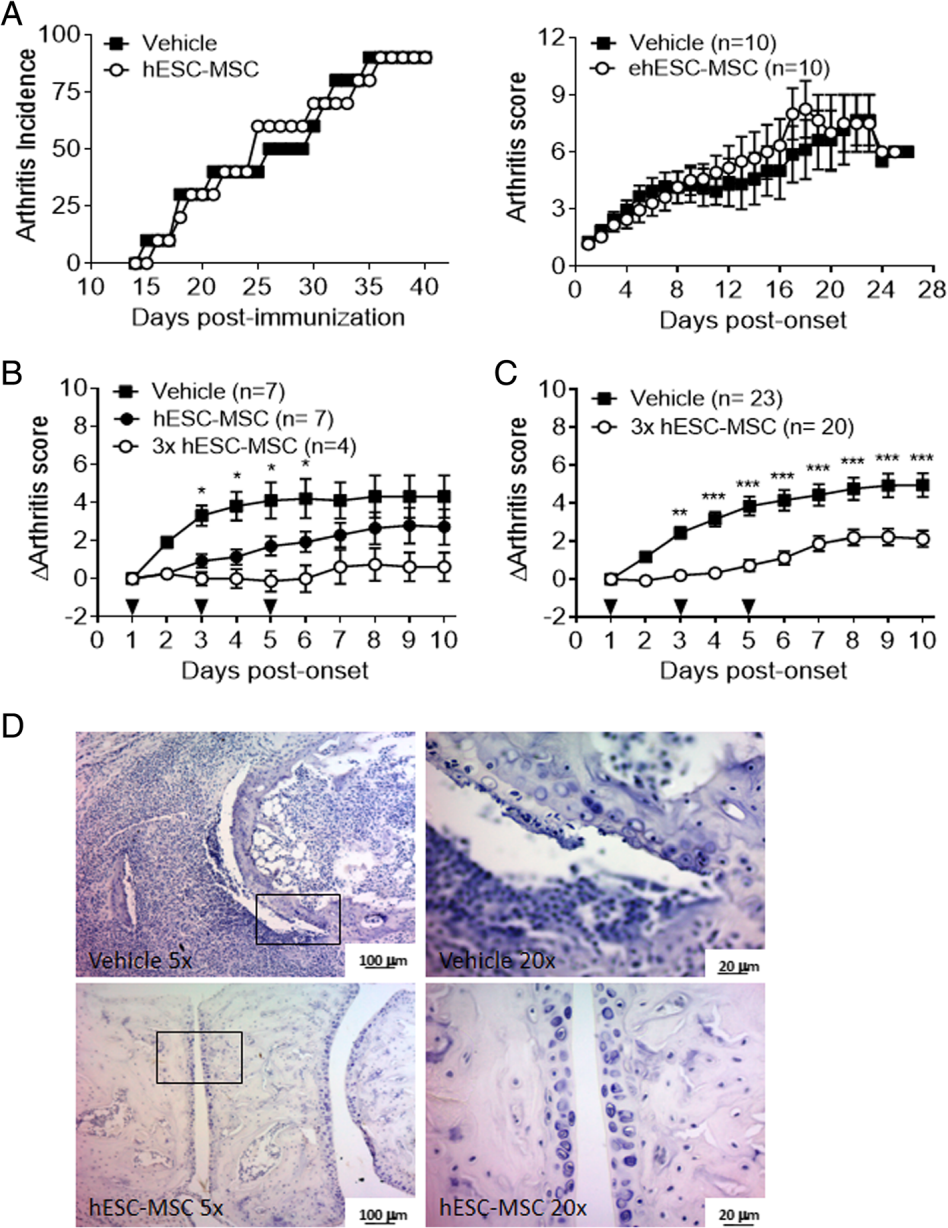
Administration of embryonic stem cell-derived mesenchymal stromal cells (*hESC-MSC*) ameliorates established collagen-induced arthritis (CIA). **a** Progression of arthritis after prophylactic administration of hESC-MSC in one experiment representative of two performed. Incidence (*left panel*) and arthritis score (*right panel*) were comparable to the control group when hESC-MSC were administered on the day of type II collagen (CII) immunization. **b** Dose-response effect of the therapeutic administration of hESC-MSC on arthritis score. Statistical differences between control and single-dose groups are indicated. **c** Amelioration of CIA induced by administration of three doses (*arrowheads*) of hESC-MSC is sustained in mice with established disease. Data pooled from three independent experiments with four to ten mice per experimental group. **d** Representative hematoxylin-eosin staining of joints after treatment with hESC-MSC in mice with established disease. *P < 0.05; **P < 0.01; ***P < 0.001

To test the ability of hESC-MSC to modulate the progression of arthritis after CII immunization, DBA/1 mice were treated prophylactically with 10^6^ cells at the time of immunization, and the evolution of arthritis was assessed daily for 6 weeks. Arthritis incidence was comparable between groups, reaching 90 % by the end of the experiment (Fig. 1a). Likewise, the severity of arthritis was comparable between experimental groups, and no protective effect was observed after prophylactic administration of hESC-MSC (Fig. 1a). These data suggest that inflammatory signals may underlie the hESC-MSC-mediated immunosuppressive response, as widely suggested [23]. To test this hypothesis, we administered hESC-MSC to DBA/1 mice starting on the day of arthritis onset (clinical score ≥1) and examined the clinical response of mice with established CIA. Treatment with a single dose of hESC-MSC (10^6^ cells) significantly reduced arthritis severity and slowed the disease progression in comparison to the control group (Fig. 1b). Disease improvement was noticed the first day after hESC-MSC infusion and was maintained up to day 6 after arthritis onset. Administration of three doses of hESC-MSC (10^6^ cells every other day) resulted in a more pronounced and significant clinical amelioration that was sustained for the duration of the experiment (Fig. 1b and c). In agreement with these observations, on histological analysis of the joints there was reduced cellular infiltration and decreased bone and cartilage destruction in hESC-MSC-treated mice compared to the control group (Fig. 1d). Together, these data suggest a robust therapeutic anti-inflammatory effect of hESC-MSC in controlling the progression of established arthritis.

### hESC-MSC induce accumulation of Treg and Th1 cells in draining lymph nodes

**Fig. 2:**
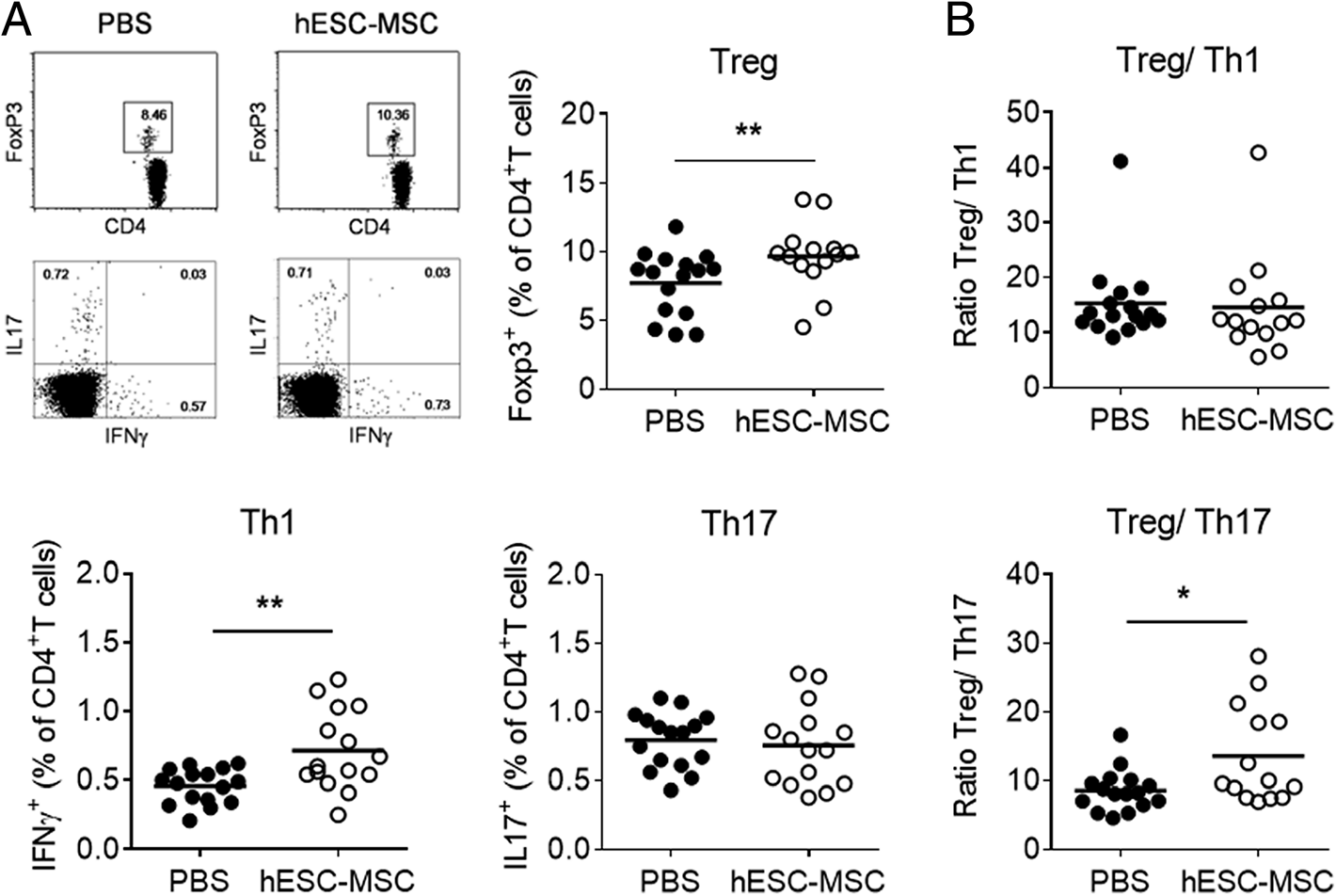
Embryonic stem cell-derived mesenchymal stromal cells (*hESC-MSC*) administration alters the regulatory T cell (*Treg*)/effector T cell (Teff) balance in mice with collagen-induced arthritis. The frequency of Treg and Teff cells was analyzed by flow cytometry in the inguinal lymph nodes (ILN) 10 days after arthritis onset in mice with established disease. **a** Representative flow cytometry plots and quantification of CD4 Treg and Teff cell frequency in hESC-MSC-treated and control mice. **b** Treg/T helper (*Th*)17 ratio is significantly increased in hESC-MSC-treated mice compared to the control group. Data shown are values from individual mice and the mean of experimental groups. Statistical differences were analyzed by the Mann-Whitney *U* test. *P < 0.05; **P < 0.01. *PBS* phosphate-buffered saline, *IFN* interferon

To characterize the mechanism underlying the protective role of hESC-MSC in CIA, we evaluated the frequency of regulatory and effector CD4^+^ T cells in draining the ILN of mice with CIA. Increased frequencies of both Treg cells (CD4^+^ Foxp3^+^) and Th1 cells (CD4^+^ IFNγ^+^) were found in the hESC-MSC-treated group compared to the PBS-treated group (Treg 9.66 ± 0.65 % vs 7.72 ± 0.58 %, *P* = 0.009; Th1 0.71 ± 0.07 % vs 0.46 ± 0.03 %, *P* = 0.007). However, no significant differences in Th17 cells (CD4^+^ IL17^+^) were detected (0.75 ± 0.07 % vs 0.79 ± 0.05 %, *P* = 0.45) (Fig. 2a). Importantly, an increased Treg/Th17 ratio was observed in the hESC-MSC with respect to the control group (13.59 ± 1.90 vs 8.58 ± 0.74, *P* = 0.04), an indicator that has been previously linked to therapeutic efficacy in human RA [24]. In contrast, no difference in Treg/Th1 ratio was found between the experimental groups (Fig. 2b).

**Fig. 3:**
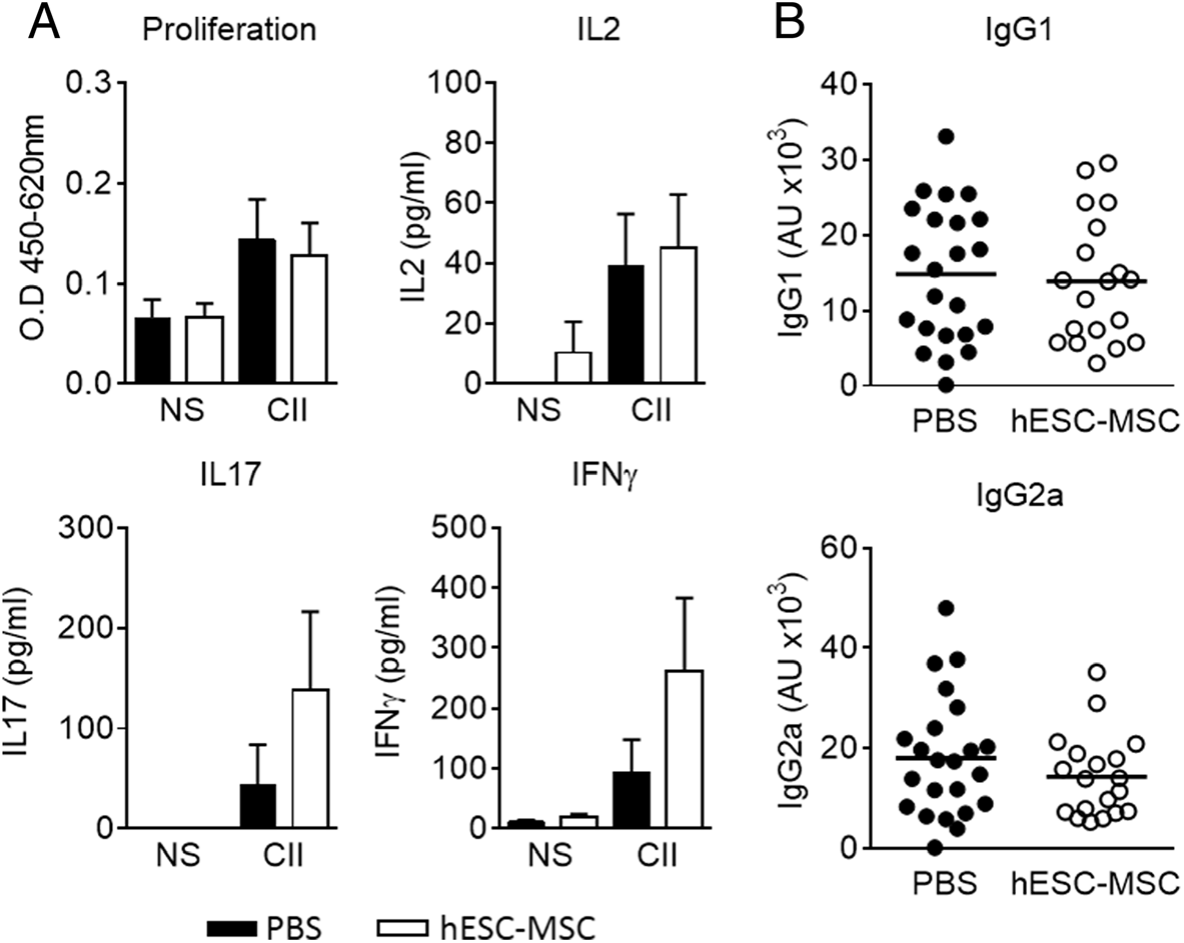
Type II collagen (*CII*)-specific T cell responses in vitro and anti-CII antibody levels in embryonic stem cell-derived mesenchymal stromal cells (*hESC-MSC*)-treated mice. **a** Proliferation and cytokine production in response to CII by inguinal lymph node cells from hESC-MSC and phosphate-buffered saline (*PBS*)-treated mice with established collagen-induced arthritis. Data represent the mean ± the standard error of the mean of the experimental groups. **b** Comparable levels of anti-CII IgG1 and IgG2a were measured in sera from control and hESC-MSC-treated mice. Data shown are values from individual mice and the mean of experimental groups. Statistical differences were analyzed by the Mann-Whitney *U* test and no significant differences were found. *NS* non-stimulated

To gain insights into the antigen specificity of the alterations observed in the homeostasis of T cell subsets, T cell proliferation and cytokine production was evaluated in the ILN upon in vitro stimulation with CII. Although there was a specific proliferative response to CII compared to non-stimulated cells (NS) (Fig. 3a), no significant differences were observed between experimental groups (hESC-MSC 0.12 ± 0.03; PBS 0.14 ± 0.04, *P* = 0.97). Accordingly, similar levels of IL2 were detected after CII stimulation (hESC-MSC 44.96 ± 17.92 pg/ml; PBS 39.13 ± 17.29 pg/ml, *P* = 0.50). Furthermore, no significant differences were observed in response to CII in the production of IL17 (138.20 ± 78.14 pg/ml in hESC-MSC-treated vs 43.37 ± 40.61 pg/ml in PBS-treated mice, *P* = 0.26) and IFNγ (261.60 ± 121.60 pg/ml in the hESC-MSC group vs 94.17 ± 53.79 pg/ml in the PBS group, *P* = 0. 67) (Fig. 3a).

Analysis of the humoral response in the sera from mice with CIA showed comparable titers of anti-CII IgG1 (13.85 ± 1.92 AU in hESC-MSC vs 14.79 ± 1.88 AU in PBS, *P* = 0.68) and IgG2a (14.29 ± 1.89 AU in hESC-MSC vs 18.05 ± 2.52 AU in PBS, *P* = 0.35) (Fig. 3b). Together, these data indicate that administration of hESC-MSC in arthritic mice promotes preferential accumulation of Treg and Th1 cells in the ILN and increases the Treg/Th17 ratio without altering the profile of anti-CII antibodies.

### hESC-MSC migrate to ILN and induce expression of indoleamine 2,3 dioxygenase

**Fig. 4:**
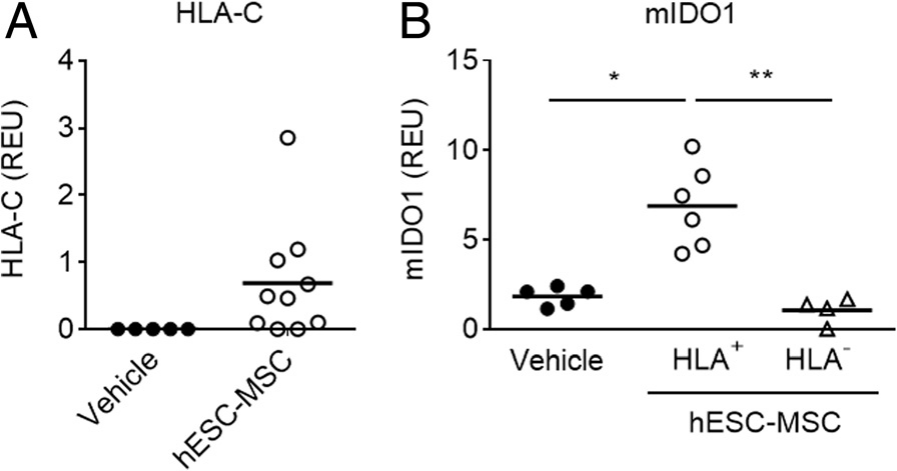
Embryonic stem cell-derived mesenchymal stromal cells (*hESC-MSC*) migrate to the inguinal lymph nodes (ILN) and induce expression of murine indoleamine 2,3 dioxygenase (IDO1). **a** Expression of HLA-C transcripts in the ILN of mice with collagen-induced arthritis (CIA) treated with hESC-MSC compared to the phosphate-buffered saline (PBS)-treated group. **b** Expression of murine IDO1 (*mIDO1*) in the ILN of mice with CIA treated with vehicle (PBS) or with hESC-MSC and showing evidence of migration (HLA^+^) or not (HLA^–^). Data show values from individual mice and the mean of the experimental groups. Statistical differences were analyzed by Kruskall-Wallis analysis of variance and Dunn’s multiple comparisons test. *P < 0.05; **P < 0.01

The observation that Treg and Th1 cell frequencies are increased in the ILN of hESC-MSC-treated mice without affecting CII-specific responses suggests that hESC-MSC migrate to the draining ILN at the site of inflammation and promote an antigen-independent immunoregulatory state. As IFNγ induces IDO1-mediated immunoregulation in bone marrow (BM)-MSC [10], we hypothesized that the accumulation of IFNγ^+^ Th1 cells in the ILN may induce the expression of IDO1 by hESC-MSC. To further explore this hypothesis, expression of human-specific HLA-C transcripts was analyzed by RT-qPCR to ascertain the presence of hESC-MSC in the paws and ILN of treated mice. Although HLA-C transcripts were not detected in the paws in any experimental condition (data not shown), they were specifically present in the ILN of most mice treated with hESC-MSC (Fig. 4a), strongly suggesting selective migration of hESC-MSC to the ILN. However, IDO1 expression of human origin (hIDO) was not detected either in the ILN of mice with expression of HLA-C trancripts or in any other experimental condition (data not shown). Surprisingly, detection of HLA-C in the ILN (HLA^+^) was associated with increased expression of mouse IDO compared to control-treated mice (*P* < 0.05) and hESC-MSC-treated non-colonized mice (HLA^–^) (*P* < 0.01) (Fig. 4b), indicating that IFNγ^+^ Th1 cells induced by hESC-MSC promote expression of mouse IDO1 by endogenous cells.

## Discussion

MSC possess broad immunoregulatory abilities and can influence both adaptive and innate immune responses. Previous studies have extensively investigated the use of adult MSC as cellular therapy in several inflammatory diseases, including RA [25, 26]. As MSC may migrate to sites of injury in vivo*,* it is reasonable to suggest that targeting the cells to inflamed joints might have a therapeutic effect on arthritis through MSC-mediated immunosuppression. Several groups have recently demonstrated the in vivo therapeutic effect of human cord-blood- and bone-marrow-derived MSC in arthritis [14, 17, 27]. In contrast, some other groups have pointed out an adverse effect when MSC are administered in mice with CIA [28, 29]. These conflicting data reinforce the need for further research into the role of MSC in chronic arthritis.

MSC derived from hPSC represent an infinite cellular source and have been successfully derived by our group upon specific inhibition of SMAD2/3 signaling [6, 21]. hESC-MSC display the same phenotype and differentiation potential as adult MSC [6, 20, 30]. Importantly, they show immunosuppressive and anti-inflammatory properties in vitro and have demonstrated a protective role in vivo in an acute model of inflammation [6].

To elucidate the effect of hESC-MSC in a chronic model of arthritis, mice with CIA were treated with hESC-MSC at different stages of arthritis development. Our results demonstrated amelioration of established arthritis after hESC-MSC infusion compared to control-treated mice (therapeutic effect). However, prophylactic administration of these cells during the induction phase of CIA did not affect disease incidence or severity. According to previous findings, the contrasting results of prophylactic vs therapeutic treatment suggest that hESC-MSC might induce differential effects depending on the time of administration and their state of activation [31–33]. In this sense, it has been demonstrated that MSC become activated in an inflammatory environment, and suppress T cell proliferation. In this scenario, MSC adopt an immune-suppressive phenotype by secreting high levels of soluble factors [23]. This could explain the anti-inflammatory effect of hESC-MSC in established arthritis and the absence of such an effect when administered prophylactically (during the induction of the disease) within a non-inflammatory environment.

Treatment with hESC-MSC altered the T cell subsets in the ILN of mice with established arthritis. Notably, there was increased frequency of Treg, in agreement with previous reports demonstrating that human adipose-derived MSC cells reduce the severity of CIA and generate Treg cells *de novo* [15]. Likewise, infusion of gingiva derived-MSC has also resulted in increased levels of CD4^+^ CD39^+^ FoxP3^+^ cells in the spleen, ILN and synovial fluid of arthritic mice [34]. Our finding that Th1 IFNγ^+^ cells are also increased in the ILN of hESC-MSC-treated mice is consistent with the protective role of IFNγ in CIA [35–37] and some reports of therapeutic use of recombinant IFNγ in human RA [38, 39]. Furthermore, elevated production of IFNγ by Th1 cells can also contribute to the elevated frequencies of Treg cells in hESC-MSC-treated mice [40].

It has been recently reported that IDO1 induction is involved in the regulation of IL17 by IFNγ [36]. In our study, mice treated with hESC-MSC had similar levels of IL17 regardless of the higher expression of IFNγ. The reason for such a discrepancy may lie in the experimental conditions used by Lee et al. to address the role of IDO1. We evaluated differentiated Th17 cells and their IL17 production in secondary lymph nodes (LN), whereas Lee et al. measured IL17 production by CD4 T cells isolated from naive mice under in vitro Th17 polarizing conditions [36]. In addition, elevated levels of IL17 production by DBA/1 mice may prove more resistant to inhibition than in the C57Bl6 mice used by Lee et al. [37].

We investigated whether hESC-MSC were able to migrate to draining lymph nodes to promote an antigen-independent immunoregulatory state. Results confirmed HLA expression in hESC-MSC-treated CIA mice, indicating migration of hESC-MSC to ILN at the end of the experiments, 5 days after the last infusion. Although we have not determined the presence of human cells in mice beyond this time point, recent evidence indicates that human MSC are not detected in the organs of mice with CIA by 10 days after administration [41], an observation that can explain the increased efficiency of repeated doses of hESC-MSC to achieve sustained amelioration of arthritis and the lack of efficacy of the prophylactic treatment.

As IFNγ and CTLA-4 expression on Treg are the main promoters of IDO1 expression, the induction of IFNγ^+^ Th1 and Treg cells by hESC-MSC infusion suggests that the immunoregulatory function of hESC-MSC might be mediated by IDO1 [42, 43]. Evidence of IDO1-dependent amelioration of CIA has been recently shown by treatment with TGF-β-induced Treg [44]. Moreover, we have previously demonstrated that IDO1 expression is induced during CIA in dendritic cells (DC) in the ILN and that genetic knockdown or pharmacological inhibition of IDO activity is associated with increased severity of CIA [45]. Conversely, administration of the IDO1 product kynurenine or synthetic analogs of tryptophan catabolites ameliorates arthritis [45, 46]. Mice with migration of hESC-MSC to the ILN had significantly higher expression of IDO1 than hESC-MSC-treated non-colonized mice or control-treated mice, indicating an IDO1-mediated immunoregulatory function of hESC-MSC in mice with CIA. Surprisingly, the source of IDO1 expression was of mouse origin and not the hESC-MSC themselves, suggesting that hESC-MSC ameliorate CIA by directly acting on the host T cells that in turn induce expression of IDO by mouse DC. This finding raises the possibility that the amelioration induced by hESC-MSC in CIA could be the result of a non-specific response to the injection of xenogeneic cells, independently of their MSC phenotype. In accordance with most reports on the therapeutic application of human MSC in CIA [19, 47, 48], our experimental setup did not include a treatment group composed of other human cell types. However, Zhou et al. have shown that administration of human cells of non-mesenchymal lineage is ineffective in the treatment of established CIA [18]. Moreover, human umbilical-cord-MSC are required to be alive to exert their therapeutic action in CIA [17]. Together, these observations make it unlikely that their xenogeneic origin plays a role in the protection triggered by hESC-MSC, and point to the induction of a specific immunoregulatory response.

In summary, our data show that hESC-MSC administration induces accumulation of Treg and Th1 cells and promotes expression of endogenous IDO1 in draining lymph nodes, thus, reducing disease progression and the severity of CIA. Therefore, hESC-MSC can provide an infinite cellular source for therapeutic interventions in RA.

## Conclusions

Administration of hESC-MSC to mice with established arthritis reduces disease severity. Injection of these cells into arthritic mice promotes accumulation of Treg and Th1 cells in the ILN, and modulates inflammation by inducing the expression of indoleamine 2,3 dioxygenase in the host.

## Abbreviations

ANOVA: analysis of variance
AU: antibody units
cDNA: complementary DNA
CFA: Complete Freund’s Adjuvant
CIA: collagen-induced arthritis
CII: type II collagen
DC: dendritic cells
DMEM: Dulbecco’s modified Eagle’s medium
ELISA: enzyme-linked immunosorbent assay
FCS: fetal calf serum
FITC: fluorescein isothiocyanate
GAPDH: glyceraldehyde-3-phosphate dehydrogenase
IFN: interferon
hESC-MSC: embryonic stem cell-derived mesenchymal stromal cells
hPSC: human pluripotent stem cells
IDO1: indoleamine 2,3 dioxygenase
ILN: inguinal lymph nodes
iPSC: induced pluripotent stem cells
MSC: mesenchymal stromal cells
PBS: phosphate-buffered saline
RA: rheumatoid arthritis
RT-qPCR: quantitative real-time polymerase chain reaction
SEM: standard error of the mean
TGF: transforming growth factor
Treg: regulatory T cells
Th: helper T cells
